# Quit attempts and cessation support among youth smokers in Saudi Arabia: A cross-sectional analysis of the 2022 Global Youth Tobacco Survey

**DOI:** 10.18332/tid/215002

**Published:** 2026-01-28

**Authors:** Moroj A. Aldarmasi

**Affiliations:** 1 Preventive Medicine and Public Health Department, Faculty of Medicine, King Abdulaziz University, Jeddah, Saudi Arabia; 2 Preventive Medicine Unit, King Abdulaziz University Hospital, Jeddah, Saudi Arabia

**Keywords:** adolescent smoking, quit attempts, tobacco cessation, Saudi Arabia, Global Youth Tobacco Survey

## Abstract

**INTRODUCTION:**

Tobacco use among adolescents continues to pose a major public-health challenge in Saudi Arabia. Despite national prevention efforts and declining smoking rates, many youths remain vulnerable to nicotine addiction and experimentation with emerging products such as shisha and e-cigarettes. Understanding factors that influence quit attempts and cessation awareness is essential to guide effective school- and community-based tobacco-control interventions. This study assessed the prevalence of quit attempts and identified behavioral and environmental correlates of cessation motivation among Saudi youth using data from the 2022 Global Youth Tobacco Survey.

**METHODS:**

A cross-sectional analysis was conducted using data from 6983 students aged 11–17 years who participated in the 2022 Global Youth Tobacco Survey. Weighted analyses described tobacco-use patterns and cessation behaviors. Chi-squared tests examined bivariate relationships, while multivariable logistic regression identified independent factors associated with quit attempts, including adjusting for age, sex, parental and peer smoking, and media exposure. Significance was defined as p<0.05.

**RESULTS:**

Approximately 33% of respondents had ever used tobacco or nicotine, and 10.8% were current users. Among those who used tobacco within the past 12 months (n=411), 77.4% had attempted to quit, 64% wanted to stop, and 79.8% had received advice to quit. Factors associated with quit attempts included having no close friends who smoke (AOR=4.38; 95% CI: 1.73–11.07), exposure to school-based anti-tobacco lessons (AOR=3.25; 95% CI: 1.51–6.99), noticing health warnings on shisha packs (AOR=2.59; 95% CI: 1.02–6.55), and exposure to tobacco imagery in media (AOR=3.19; 95% CI: 1.64–6.17).

**CONCLUSIONS:**

Most Saudi youth who use tobacco express a desire to quit, and social context strongly influences their cessation behavior. Reinforcing school-based anti-tobacco education, expanding adolescent cessation programs, and strengthening policy enforcement could further reduce tobacco use and improve cessation outcomes among young people.

## INTRODUCTION

Tobacco use remains one of the most preventable causes of morbidity and premature death worldwide^1^. Despite advances in global tobacco-control policies, adolescent tobacco and nicotine use continues to rise in many developing regions, driven by social influence, product diversity, and limited access to cessation support^2,3^. Adolescents are particularly vulnerable to nicotine addiction because early exposure and developmental susceptibility increase their risk of more severe dependence and long-term use into adulthood^4-6^.

In Saudi Arabia, national surveillance reports have shown a gradual decline in conventional cigarette smoking but a growing preference for alternative products such as shisha and e-cigarettes^7-10^. These shifting patterns highlight the changing landscape of youth tobacco use, shaped by social acceptability, peer pressure, and online marketing^7,11,12^. Early initiation of tobacco use is linked to higher lifetime dependence and greater difficulty in quitting later in life^13-15^.

Quit attempts among adolescents offer insight into the effectiveness of ongoing tobacco-control strategies. Studies conducted in the Gulf Cooperation Council region show that many youths express a desire to quit but face barriers including peer smoking, parental influence, and lack of structured cessation services^12,16-19^. In contrast, protective factors such as anti-tobacco education, awareness of health warnings on tobacco packaging, and effective smoke-free policies are associated with higher quit motivation and quit-attempt rates among adolescents^8,11,20-22^.

However, evidence specific to Saudi adolescents remains limited, particularly in identifying factors associated with quit attempts across behavioral and environmental domains. The 2022 Global Youth Tobacco Survey provides a timely opportunity to explore these factors at a national level. This study aimed to assess the prevalence of quit attempts and identify determinants of cessation motivation and support among youth who reported use of any tobacco or nicotine product (cigarettes, shisha, or e-cigarettes) in Saudi Arabia, providing evidence to inform future school-based and community-level cessation initiatives.

## METHODS

### Study design and data source

This study utilized a cross-sectional design based on data from the 2022 Global Youth Tobacco Survey (GYTS) conducted in Saudi Arabia. The GYTS is a nationally representative, school-based survey designed to monitor tobacco use, exposure, and cessation behaviors among students aged 11–17 years. It employs a standardized two-stage cluster sampling method to ensure national representativeness. The survey instrument follows the World Health Organization and U.S. Centers for Disease Control and Prevention core methodology. A formal participant flow diagram was not generated because the analysis was based on a single cross-sectional survey dataset with predefined sampling and inclusion criteria.

### Study population

The analytic sample included 6983 students who provided complete data on tobacco use and cessation-related variables. Participants were categorized as ‘current users’ if they reported use of any tobacco or nicotine product (cigarettes, shisha, or e-cigarettes) within the past 30 days. A subset of students who reported tobacco or nicotine use within the past 12 months was analyzed to determine quit-attempt behaviors. Of the total 6983 surveyed students, 411 respondents who reported current use of any tobacco or nicotine product and had complete data on quit attempts were included in the final analytic sample.

### Measures and variables

The primary outcome variable was self-reported quit attempt (yes, no) among users of any tobacco or nicotine product. Independent variables included demographic characteristics (age, sex, grade, weekly allowance), parental and peer smoking status, exposure to anti-tobacco education, exposure to health warnings, and media visibility of tobacco use. Awareness of cessation services and receipt of advice to quit were also examined as secondary outcomes.

### Statistical analysis

All analyses were performed using complex-sample procedures to account for the multistage cluster sampling design of the GYTS. Survey sampling weights, primary sampling units, and strata were applied in all descriptive, bivariate, and multivariable logistic regression analyses to ensure national representativeness. Descriptive statistics were used to summarize sociodemographic characteristics and tobacco-use patterns. Chi-squared tests were used to assess bivariate associations between quit attempts and associated factors. Variables showing p<0.10 in bivariate analysis were entered into a multivariable logistic regression model to identify factors associated with quit attempts, adjusting for potential confounders (age, sex, parental smoking, and peer influence). Model goodness-of-fit was assessed using the Hosmer–Lemeshow test. Potential interaction terms (sex × age group) were examined and found not to be statistically significant; therefore, no interaction terms were retained in the final model. Adjusted odds ratios (AOR) with 95 % confidence intervals (CIs) were reported, and statistical significance was set at p<0.05. All statistical tests were two-tailed. Missing data were handled using complete-case analysis, as the proportion of missing responses for key variables was minimal.

### Ethical considerations

The study was approved by the Unit of Biomedical Ethics at King Abdulaziz University Hospital (Reference No 459-25). The GYTS protocol adhered to the ethical principles outlined in the Declaration of Helsinki. Participation was anonymous and voluntary, and no identifying information was collected from respondents or schools. Written informed consent was obtained from school authorities and parents or legal guardians, and verbal assent was obtained from all participating students before data collection.

## RESULTS

### Participant characteristics

A total of 6983 students aged 11–17 years were included in the analysis. Slightly more than half were male (52.4 %), and the majority (58.4 %) were aged between 13–14 years. About one-third (32.9 %) reported having ever used a tobacco or nicotine product, and 10.8 % were current users (Tables 1 and 2). Most respondents (79.9 %) reported no parental smoking, and 83.7 % stated that none of their closest friends smoked.

**Table 1:** Sociodemographic and tobacco-use characteristics of GYTS study participants in Saudi Arabia, 2022 (N=6983)

*Characteristics*	*n*	*%*
**Age** (years)		
≤11	130	1.9
12	700	10.1
13	1928	27.7
14	2140	30.7
15	1542	22.1
16	417	6.0
≥17	105	1.5
**Sex**		
Male	3615	52.4
Female	3283	47.6
**Grade**		
1st Intermediate	2363	34.3
2nd Intermediate	2433	35.3
3rd Intermediate	2096	30.4

Percentages are weighted to national representation.

**Table 2:** Tobacco and nicotine product use and economic characteristics of study participants, GYTS Saudi Arabia, 2022 (N=6983)

*Characteristics*	*n*	*%*
**Weekly allowance** (SAR)		
None	1589	23.0
<30	2470	35.7
30–49	1344	19.4
50–99	888	12.8
≥100	624	9.0
**Parental smoking status**		
None	5560	79.9
Father only	824	11.8
Mother only	48	0.7
Both parents	279	4.0
Don’t know	244	3.5
**Closest friends who smoke**		
None	5810	83.7
Some	824	11.9
Most	229	3.3
All	79	1.1
**Ever used any tobacco/nicotine product**	2297	32.9
**Any product use in past 30 days**	754	10.8

SAR: 100 Saudi Arabian Riyals about US$270.

### Cessation awareness and quit attempts

**Figure 1:**
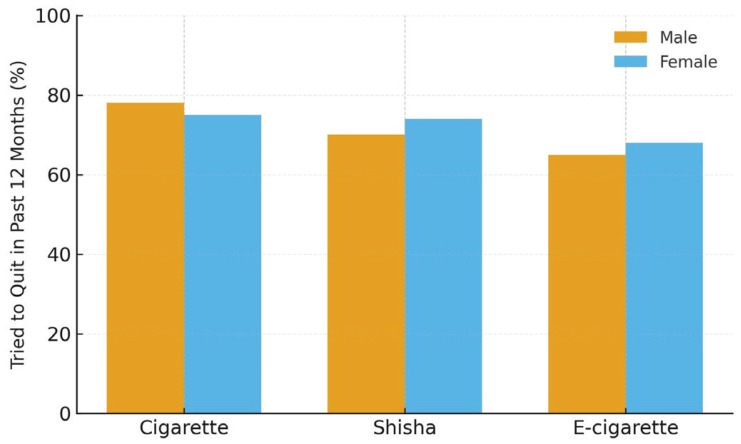
Quit attempts among youth users of cigarettes, shisha, or e-cigarettes in the past 12 months, Saudi Arabia, 2022 (N= 411)

Among respondents who reported tobacco or nicotine use within the past 12 months (n=411), 77.4% reported at least one quit attempt, 64% expressed a desire to stop, and 79.8% had received some form of advice or cessation support (Table 2). Most youth reported confidence in their ability to quit, indicating favorable motivation toward cessation. Males were slightly more likely to attempt quitting cigarettes, whereas females reported more cessation attempts for shisha and e-cigarettes (Figure 1) (Table 3).

**Table 3:** Quit attempts and cessation awareness among youth users of any tobacco or nicotine product in the past 12 months, GYTS, Saudi Arabia, 2022 (N=411)

*Indicator*	*n*	*%*
**Tried to quit in past 12 months**		
Yes	318	77.4
No	93	22.6
**Wants to stop now** (N=361)		
Yes	231	64.0
No	130	36.0
**Believes able to stop** (N=262)		
Yes	191	72.9
No	71	27.1
**Ever received cessation help/advice** (N=540)		
Yes	431	79.8
No	109	20.2

### Bivariate associations

As shown in Table 4, significant differences in quit-attempt rates were observed according to peer and environmental exposures. Students whose closest friends smoked were markedly less likely to have attempted quitting compared with those whose friends did not smoke. Support for smoke-free public places and exposure to anti-tobacco messages were also positively associated with quit attempts.

**Table 4:** Bivariate associations between factors and quit attempts in students aged 11–17 years who participated in the GYTS Saudi Arabia, 2022 (N=6983)

*Predictor*	*Category*	*Total* *n*	*Quit attempt*			p*
			*Yes* *n*	*No* *n*	*Yes* *%*	
**Closest friends smoke**	None	86	60	26	69.8	0.226
	Some	110	90	20	81.8	
	Most	39	30	9	76.9	
	All	34	22	12	64.7	
**Favor ban in enclosed public places**	Yes	177	146	31	82.5	0.007
	No	100	68	32	68.0	
**Refused sale of e-cigarettes due to age** (past 30 days)	Did not try to buy	143	119	24	83.2	0.043
	Yes	48	33	15	68.8	
	No	93	67	26	72.0	
**Saw tobacco in TV/movies** (past 30 days)	Did not watch	69	53	16	76.8	<0.001
	Yes	156	132	24	84.6	
	No	65	39	26	60.0	

* χ^2^ test used for bivariate comparisons; no missing data imputation.

### Multivariable analysis

In the weighted multivariable logistic regression model (Table 5), factors associated with quit attempts included having no close friends who smoke (AOR=4.38; 95% CI: 1.73–11.07), exposure to classroom-based anti-tobacco education (AOR=3.25; 95% CI: 1.51–6.99), noticing health warnings on shisha packs (AOR=2.59; 95% CI: 1.02–6.55), and exposure to people using tobacco in movies or television (AOR=3.19; 95% CI: 1.64–6.17). These associations remained significant after adjusting for age, sex, and parental smoking. The overall model fit was satisfactory (Hosmer–Lemeshow p>0.05).

**Table 5:** Weighted multivariable logistic regression of factors associated with quit attempts among youth users of any tobacco or nicotine product, GYTS Saudi Arabia, 2022 (N=411)

*Predictor*	*AOR*	*95% CI*	*p*
Closest friends smoke: None (vs …)	4.38	1.73–11.07	0.002
Closest friends smoke: Some (vs …)	2.90	1.16–7.22	0.022
Anti-tobacco warnings on shisha led to think about quitting	2.59	1.02–6.55	0.045
Taught in class about dangers of tobacco: Yes (vs Don’t know)	3.25	1.51–6.99	0.003
Saw people using tobacco in TV/movies: Did not watch (vs No)	2.46	1.06–5.71	0.036
Saw people using tobacco in TV/movies: Yes (vs No)	3.19	1.64–6.17	0.001

AOR: adjusted odds ratio. Binary logistic regression (backward conditional method) adjusted for age, sex, parental smoking, and media exposure. All odds ratios were derived from weighted logistic regression models accounting for the complex GYTS sampling design. Model fit was assessed satisfactory using the Hosmer–Lemeshow goodness-of-fit test (p>0.05). No significant interaction between age and sex was identified.

**Figure 2:**
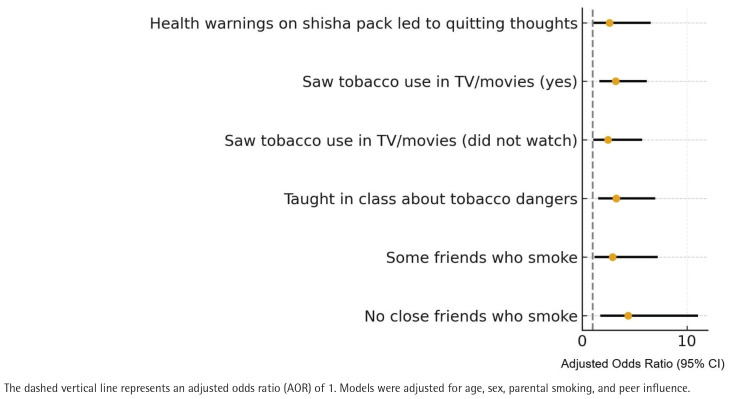
Adjusted odds ratios for factors associated with quit attempts among current youth use of any tobacco or nicotine product in Saudi Arabia

Collectively, the results suggest that most Saudi youth who use tobacco demonstrate cessation interest and have made at least one attempt to quit. Peer smoking, educational exposure, and awareness of health warnings emerged as factors associated with quit attempts (Figure 2).

## DISCUSSION

### Principal findings

This study provides nationally representative evidence on cessation behaviors and correlates of quit attempts among youth smokers in Saudi Arabia. More than three-quarters of adolescent users reported at least one quit attempt, and nearly two-thirds expressed a desire to stop. These findings indicate a substantial level of motivation to quit despite the continued presence of social and environmental risk factors. The high proportion of youth who received cessation advice underscores growing exposure to anti-tobacco messaging but also highlights a need for structured, youth-specific cessation services^1,23,24^. The observed quit-attempt rate (77.4%) exceeds that reported in earlier regional surveys, which ranged from 45% to 65% among Gulf adolescents. This pattern may reflect the expansion of school-based prevention initiatives and the enforcement of smoke-free policies over the past decade.

The prevalence of quit attempts observed in this study is higher than rates reported in earlier Saudi and Gulf surveys, which ranged rates ranging from 45% to 65%^12,22,25,26^. This difference may be related to intensified school-based prevention and the broader enforcement of smoke-free legislation over the past decade^22,27,28^. Consistent with current evidence, peer influence remains a dominant factor shaping adolescent quit behavior^12,22,29^. Adolescents with non-smoking friends were more than four times as likely to attempt quitting, mirroring findings from studies in Jordan and the United Arab Emirates^12,18,22,30^.

Males in this study were slightly more likely than females to report cigarette-related quit attempts, consistent with regional data showing that boys tend to initiate smoking earlier and are more likely to use conventional cigarettes, while girls report higher shisha use.

Exposure to anti-tobacco education in schools and health warnings on shisha products were also strong correlates of quit attempts, aligning with evidence that educational and visual interventions are associated with improved cessation readiness^20,31^. This supports earlier findings that structured school-based programs and visual health warnings significantly increase awareness and quit motivation among youth. Conversely, exposure to tobacco use in media remained a significant yet paradoxical predictor – possibly reflecting awareness induced by media depictions rather than promotion of use^32,33^.

Collectively, these results are consistent with the WHO MPOWER (Monitor, Protect, Offer help, Warn, Enforce, Raise taxes) framework particularly the ‘Offer help’ and ‘Warn’ components, emphasizing that educational exposure and health-warning visibility are associated with youth cessation behaviors.

These findings reinforce the need to strengthen the ‘Offer help’ component of the WHO MPOWER framework within the Saudi context^12,22,34,35^. Integrating cessation modules into the school curriculum and ensuring continuity through primary-care services may enhance adolescent quit attempts. Expanding national quitline accessibility, developing digital cessation tools, and training educators in motivational interviewing could further close the gap between willingness to quit and sustained abstinence^34,36-38^.

Peer-based interventions may be particularly promising, as they target the social networks most influential during adolescence. Programs that combine interactive education, parental engagement, and visible health warnings have demonstrated measurable reductions in youth tobacco use in other middle-income settings^39-41^.

### Limitations

The use of a nationally representative dataset and standardized WHO GYTS methodology strengthens the external validity and generalizability of these findings to school-attending adolescents in Saudi Arabia. However, the study is subject to several limitations. Tobacco and nicotine use, quit attempts, and exposure measures were self-reported, which may have introduced recall and social-desirability bias, as well as possible misclassification of exposure or outcome status. The cross-sectional design precludes causal inference and limits conclusions to associations. In addition, although key confounders such as age, sex, parental smoking, and peer influence were adjusted for, residual confounding from unmeasured factors (e.g. parental education, household income, and mental health status) cannot be excluded. Finally, because the survey included only in-school adolescents, the results may not be fully generalizable to out-of-school youth.

## CONCLUSIONS

Most Saudi adolescents who use tobacco express a strong desire to quit and have already made one or more quit attempts. Peer dynamics, exposure to anti-tobacco education, and the visibility of health warnings significantly influence their cessation behavior. The social context – particularly peer behavior – remains a major determinant of motivation to quit, while school-based education and clear health warnings strengthen quit attempts. These findings suggest that integrating tailored, youth-focused cessation support within schools and community settings, may strengthen tobacco-control efforts. Incorporating structured programs, digital tools, and peer-led interventions into the national tobacco-control framework warrants further evaluation through longitudinal and intervention-based studies to sustain progress toward reducing adolescent nicotine dependence and achieving national tobacco-control targets.

## Data Availability

The data supporting this research are available from the following sources: https://www.gtssacademy.org/explore/country/Saudi%2BArabia/Saudi%2BArabia%2B-%2BNational/survey-data/?utm_source=
